# TREM2^+^ macrophages accumulate in childhood IgA nephropathy and soluble TREM2 represents a reliable non‐invasive biomarker

**DOI:** 10.1113/EP092716

**Published:** 2025-05-05

**Authors:** Ling Yu, Xuan Gang, Jingjing Wang, Guoping Huang, Qiuyu Li, Weizhong Gu, Haidong Fu, Jianhua Mao

**Affiliations:** ^1^ Department of Nephrology Children's Hospital, Zhejiang University School of Medicine, National Clinical Research Center for Child Health Hangzhou China; ^2^ Department of Pathology Children's Hospital, Zhejiang University School of Medicine, National Clinical Research Center for Child Health Hangzhou China

**Keywords:** biomarker, childhood IgA nephropathy, macrophage, soluble TREM2, TREM2

## Abstract

IgA nephropathy (IgAN) is a common type of primary glomerulonephritis in children. The pathogenesis of childhood IgAN remains unclear, and there is a lack of effective non‐invasive biomarkers for this disease. Single‐cell RNA sequencing was performed in children with IgAN to delineate cellular and molecular compositions, and subcluster analysis for macrophages was conducted. Blood samples were collected from 38 children with IgAN to measure soluble TREM2 (sTREM2) and soluble CD163 (sCD163) levels and analyse their clinical significance. Single‐cell RNA sequencing identified distinct cell clusters in both parenchymal and stromal compartments. Mesangial components were classified into vascular smooth muscle cells/pericytes, mesangial cells, fibroblasts and activated myofibroblasts. Patients with IgAN had a marked increase in myofibroblasts and immune cells in comparison to the control group. Remarkable infiltration of macrophages was observed in the kidneys of IgAN patients, and a subgroup of marcophages with high TREM2 expression was enriched. Children with IgAN exhibited significantly higher plasma sTREM2 levels than healthy individuals, and the sTREM2 level was correlated with sCD163 abundance. Importantly, an increased sTREM2 level was positively associated with the severity of proteinuria. Moreover, the elevation of sTREM2 was correlated with a more advanced pathological grading. In summary, we unveiled a remarkable remodelling of the stromal cellular landscape in childhood IgAN, and TREM2^+^ macrophages were found to accumulate. We identified that the plasma sTREM2 level was associated with clinical and pathological severity and therefore constituted a potential non‐invasive biomarker for children with IgAN.

## Abstract (highlights)

- **What is the central question of this study?**
  The evolution of cellular composition during progression of childhood IgA nephropathy (IgAN) is complex and remains unclear. There is a lack of an efficient non‐invasive biomarker for childhood IgAN.
- **What is the main finding and its importance?**
  Using single‐cell RNA sequencing, we revealed the cellular landscape of childhood IgAN by including distinct subpopulations among mesangial‐like cells and macrophages. A subgroup of TREM2^+^ macrophages accumulated in the kidney of childhood IgAN patients. The soluble TREM2 level was correlated with the clinical and pathological severity of disease, serving as a reliable non‐invasive biomarker for IgAN.

## 1 INTRODUCTION

IgA nephropathy (IgAN) is a common type of primary glomerulonephritis and a leading cause of chronic kidney disease worldwide (Cheung et al., 2025). Although mostly being diagnosed in adults in their second or third decades of life, IgAN can also be found in children. Clinical manifestation and disease course vary for IgAN patients, with >50% developing kidney failure within 20–30 years after diagnosis (D'Amico, 2004; Pitcher et al., 2023). Standard treatment modalities include renin–angiotensin system blockade and immunosuppressive therapies, such as corticosteroids and cyclophosphamide (Lai et al., 2016). New therapies and approaches are under intensive investigation, along with deep mechanistic understanding of disease pathogenesis (Cheung et al., 2025). Currently, the assessment of disease progression, prognosis and treatment response relies largely on histological analysis, such as the Oxford classification (Trimarchi et al., 2017). There remains a lack of efficient non‐invasive biomarkers validated for clinical use in IgAN.

Pathogenesis of IgAN is complex. A ‘multi‐hit’ hypothesis has been well characterized, encompassing an increase of circulating galactose deficient‐IgA1 (Gd‐IgA1), formation of Gd‐IgA1 immune complexes with reactive antibodies, and deposition of these complexes in the kidney, with resultant complement system activation, inflammation, glomerular damage and fibrosis (Cheung et al., 2025). Once deposited in the kidney, Gd‐IgA1 immune complexes trigger aberrant activities in different types of cells, involving mesangial cells, fibroblasts, podocytes, endothelial cells and immune cells. Abundant infiltration of immune cells in glomerular and interstitial compartments has been found in IgAN. Among them, macrophages compose the major population and are associated with disease progression. Several lines of evidence have suggested that the intensity of macrophage infiltration can be used as biomarker in evaluating pathological severity and predicting response to immunosuppressive therapeutics (Xie et al., 2021; Yang et al., 2021). Nonetheless, macrophages are known to be a heterogeneous population, and each subgroup has a distinct phenotype and functional impact. Thus, delineating the molecular function of macrophage subpopulations is significant for identifying more useful and efficacious biomarkers for IgAN.

Triggering receptors expressed on myeloid cells (TREMs) are an immunoglobulin superfamily of cell‐surface receptors mainly detected on myeloid cells, such as macrophages (Colonna, 2023). TREM2 is an important component and mediates the response to tissue injury and stress (Colonna, 2023; Deczkowska et al., 2020). Functionally, TREM2 on macrophages is responsible for maintaining cell survival, promoting efferocytosis and modulating inflammation (Deczkowska et al., 2020). Many studies have shown that TREM2 plays a crucial role in a variety of diseases, including neurodegenerative diseases, cancer and metabolic pathologies (Katzenelenbogen et al., 2020; Ma et al., 2024; Patterson et al., 2023; Ulland et al., 2017). Stimulating or blocking TREM2 activities have been demonstrated to be therapeutic strategies for Alzheimer's disease and malignancy, respectively (Binnewies et al., 2021; Schlepckow et al., 2023). Moreover, cleavage of membrane TREM2 and alternative splicing generate soluble TREM2 (sTREM2), rendering it a potential non‐invasive biomarker with diagnostic and prognostic significance (Filipello et al., 2022; Indira Chandran et al., 2023).

Here, by using single‐cell transcriptomic profiling, we found that a subset of TREM2^+^ macrophages accumulated in kidneys of IgAN in comparison to healthy individuals, which is likely to be involved in the progression of this inflammatory disease. Importantly, sTREM2 in peripheral blood was shown to be correlated with pathological severity of IgAN and is potentially a useful biomarker, which warrants further validation in large cohorts.

## 2 MATERIALS AND METHODS

### 2.1 Ethical approval

Human tissue and blood samples were harvested at the Children's Hospital, Zhejiang University School of Medicine. Institutional review board approval (#KY‐2021‐0326) was obtained from the local Ethical Committee of the Children's Hospital, Zhejiang University School of Medicine, in compliance with the *Declaration of Helsinki*. Written informed consent was obtained from all individuals.

### 2.2 Patients and sample collection

For single‐cell RNA sequencing (scRNA‐seq), fresh kidney samples were collected by biopsy from two patients with IgAN at the Department of Nephrology, Children's Hospital of Zhejiang University School of Medicine, and control specimens were harvested from normal kidneys of two children with benign renal tumours undergoing surgical resection. Blood samples were collected between January 2022 and December 2023 from 38 IgAN patients and 18 healthy individuals at the same hospital. The initial processing of blood samples was completed within 6 h of sampling, and plasma was extracted for frozen storage. Data on clinical characteristics were recorded, including age, sex, quantification of 24 h proteinuria, proteinuria/creatinine ratio, serum creatinine and estimated glomerular filtration rate. Written informed consent was obtained from all individuals. The protocol on human sample processing was approved by the Institutional Review Board of our centre.

### 2.3 Single‐cell RNA sequencing, data processing and analysis

A single‐cell suspension was first prepared by mechanical mincing and digestion with collagenase. Cell concentration and viability were assessed by an automated cell counter. Chromium Controller and Single Cell 3′ Reagent kits (v.3.1) were used to capture single cells and prepare libraries according to the manufacturer's protocol. Briefly, cell suspensions were loaded onto the Chromium Single Cell Controller Instrument to generate single‐cell gel beads in emulsions (GEMs), then reverse transcription reactions were performed. Silane magnetic beads were used to remove leftover biochemical reagents and primers from the post‐GEM reaction mixture. Full‐length, barcoded complementary DNA was next amplified by PCR for library construction, which was sequenced on a NovaSeq 6000 platform (Illumina, San Diego, CA, USA). Sequencing data were aligned to the human reference genome (GRCh38) and processed using CellRanger v.2.1.1 (10X Genomics).

Low‐quality cells with expressed genes <500 and expressing >70% mitochondrial reads were removed, and there was no filtering limit on unique molecular identifier (UMI) counts. Cells were projected using the T‐distributed stochastic neighbour embedding or uniform manifold approximation and projection method. Briefly, gene expression values were calculated using the LogNormalize method of Seurat's normalization function. Principal component analysis was performed using the normalized expression values. Clustering and cell identity were defined by using the FindClusters function according to the top 20 principal components. Differentially expressed genes (DEGs) were detected by the FindAllMarkers function in Seurat, using |FC| > 2 and an adjusted *p*‐value of <0.05 as the cut‐off values. Gene Ontology (GO) and Kyoto encyclopedia of genes and genomes enrichment analysis on DEGs of each cluster were performed by using hypergeometric testing. Developmental trajectories of mesangial‐like cells were inferred using Monocle2. All analysis for scRNA‐seq data were performed using the OmicStudio tools created by LC‐BIO Co., Ltd (HangZhou, China) at https://www.omicstudio.cn/cell.

### 2.4 ELISA for measuring plasma sTREM2 and sCD163 levels

sTREM2 (ab224881; Abcam) and sCD163 (ab274394; Abcam) abundances in plasma samples were analysed by using human ELISA kits according to the manufacturer's instructions. Briefly, 50 µL of each standard and sample was added to appropriate wells, followed by the addition of 50 µL of antibody cocktail, and the mixture was incubated at room temperature for 1 h. After washing, 100 µL of development solution was added to all wells and incubated for 10 min. Approximately 100 µL of stop solution was added to all wells, followed by the measurement of absorbance at 450 nm in an ultraviolet spectrophotometer.

### 2.5 Histological staining and analysis

Biopsied kidney samples were paraffin embedded, and sections were subjected to Haematoxylin and Eosin or Periodic Acid–Schiff histological staining. Pathological lesions were graded according to Oxford's MEST‐C classification by two independent pathologists, which is based on evaluation of mesangial hypercellularity (M), endocapillary hypercellularity (E), segmental glomerulosclerosis (S), tubular atrophy/interstitial fibrosis (T), and crescents (C).

### 2.6 Immunofluorescence analysis

Biopsied kidney tissues were fixed with 10% neutral buffered formalin and embedded in paraffin. The blocks were sectioned and deparaffinized. Slides were incubated with primary antibodies and secondary antibodies sequentially for immunostaining according to standard protocol. The primary antibodies used included anti‐CD3 (1:50, ab135372; Abcam), anti‐CD68 (1:50, ab955; Abcam) and anti‐TREM2 (1:200, 91068; CST). Each slide was assessed in five high‐power field images using the Vectra imaging system (PerkinElmer).

### 2.7 Statistics

Continuous variables of plasma sTREM2 and sCD163 levels were expressed as mean values and SD, and compared with Student's unpaired independent *t‐*test or the Mann–Whitney *U*‐test between the two groups. Correlation analysis was performed using the non‐parametric Spearman correlation method. In all analyses, a two‐tailed *p*‐value of <0.05 was considered statistically significant. Statistical analyses were performed using GraphPad Prism software v.9.1.

## 3 RESULTS

### 3.1 Cellular composition for kidneys of childhood IgAN at single‐cell resolution

**FIGURE 1:**
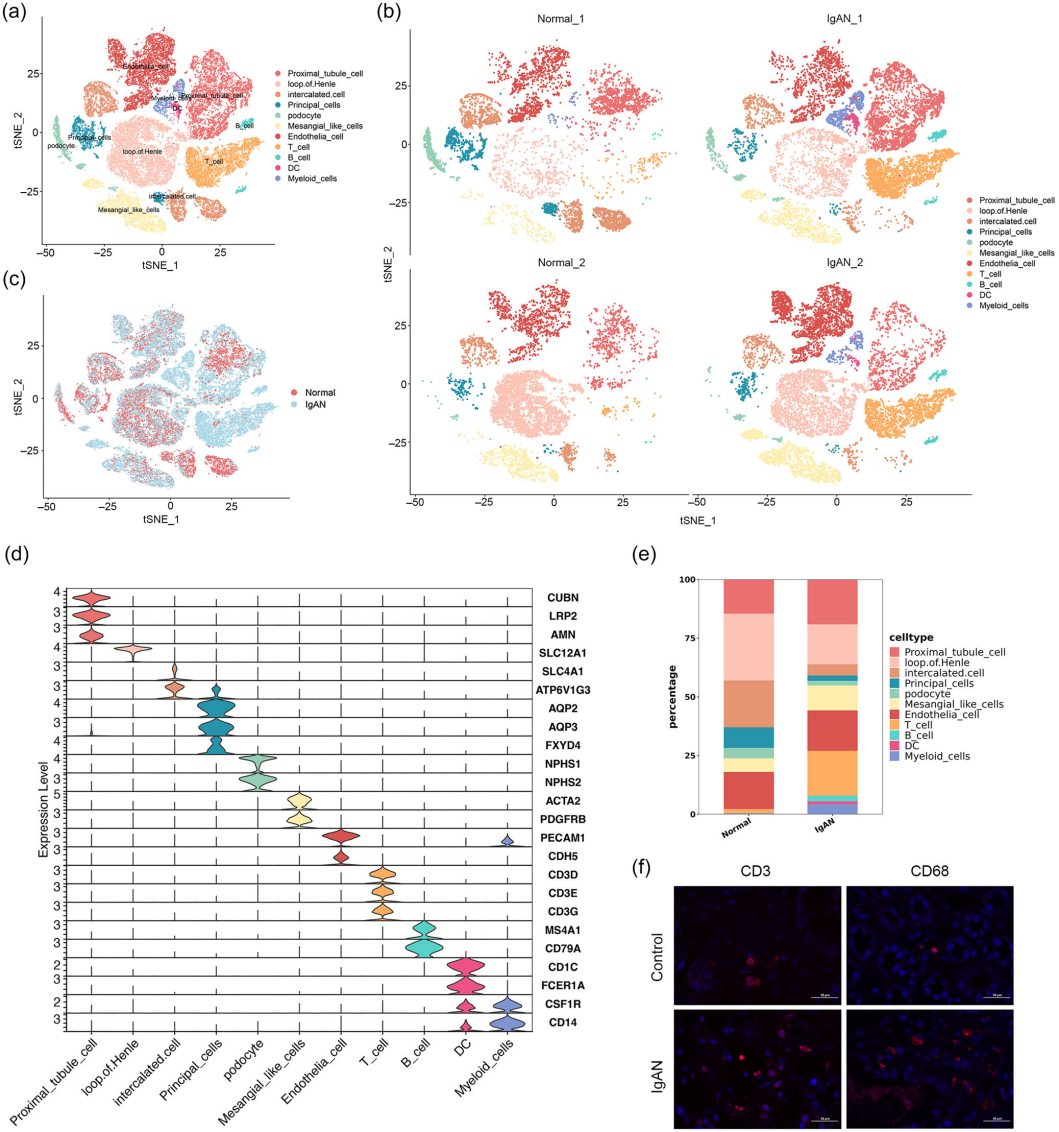
Single‐cell transcriptomic profiling for childhood IgAN. (a) T‐SNE plot showing the distinct clusters of cells in the IgAN (*n* = 2) and control (*n* = 2) groups. (b) T‐SNE plot for individual samples. (c) T‐SNE plot of clusters for IgAN patients versus normal controls. (d) Violin plots depicting the expression of selected marker genes for each cluster. (e) Comparison of percentage for cell subgroups in the IgAN versus control group. (f) Immunofluorescence staining for CD3 and CD68. Scale bars: 50 µm. Abbreviations: IgAN, IgA nephropathy; T‐SNE, T‐distributed stochastic neighbour embedding.

To profile the cellular landscape of IgAN, scRNA‐seq was performed for kidney tissues from two IgAN patients and two healthy individuals. Diagnosis of IgAN was confirmed by characterized histological alterations and positive staining for immunoglobulin protein (Figure S1). In total, 36 829 single cells were acquired and sequenced, including 7744 (Normal_1), 7219 (Normal_2), 11 597 (IgAN_1) and 10 269 (IgAN_2) cells in each sample. Eleven distinct clusters were annotated according to the canonical marker genes, including *CUBN* ^+^ *LRP2* ^+^ proximal tubular cells, *UMOD* ^+^ *SLC12A1* ^+^ loop of Henle cells, *SLC4A1* ^+^ *ATP6V1G3* ^+^ intercalated cells, *AQP2* ^+^ *AQP3* ^+^ principal cells, *NPHS1* ^+^ *NPHS2* ^+^ podocytes, *PDGFRB* ^+^ *ACTA* ^+^ mesangial cells/fibroblasts, *PECAM1* ^+^ *CDH5* ^+^ endothelial cells, *CD3* ^+^ T cells, *CD79A* ^+^ *MS4A1* ^+^ B cells, *CD1C* ^+^ dendritic cells and *CD14* ^+^ myeloid cells (Figure 1a,b). There were remarkable differences in the cellular landscape between IgAN and control groups (Figure 1c,d). In particular, immune cells were found to be substantially accumulated in children with IgAN (Figure 1c,d). Immunostaining analysis validated upregulation of markers for T cells and macrophages (Figure 1e).

**FIGURE 2:**
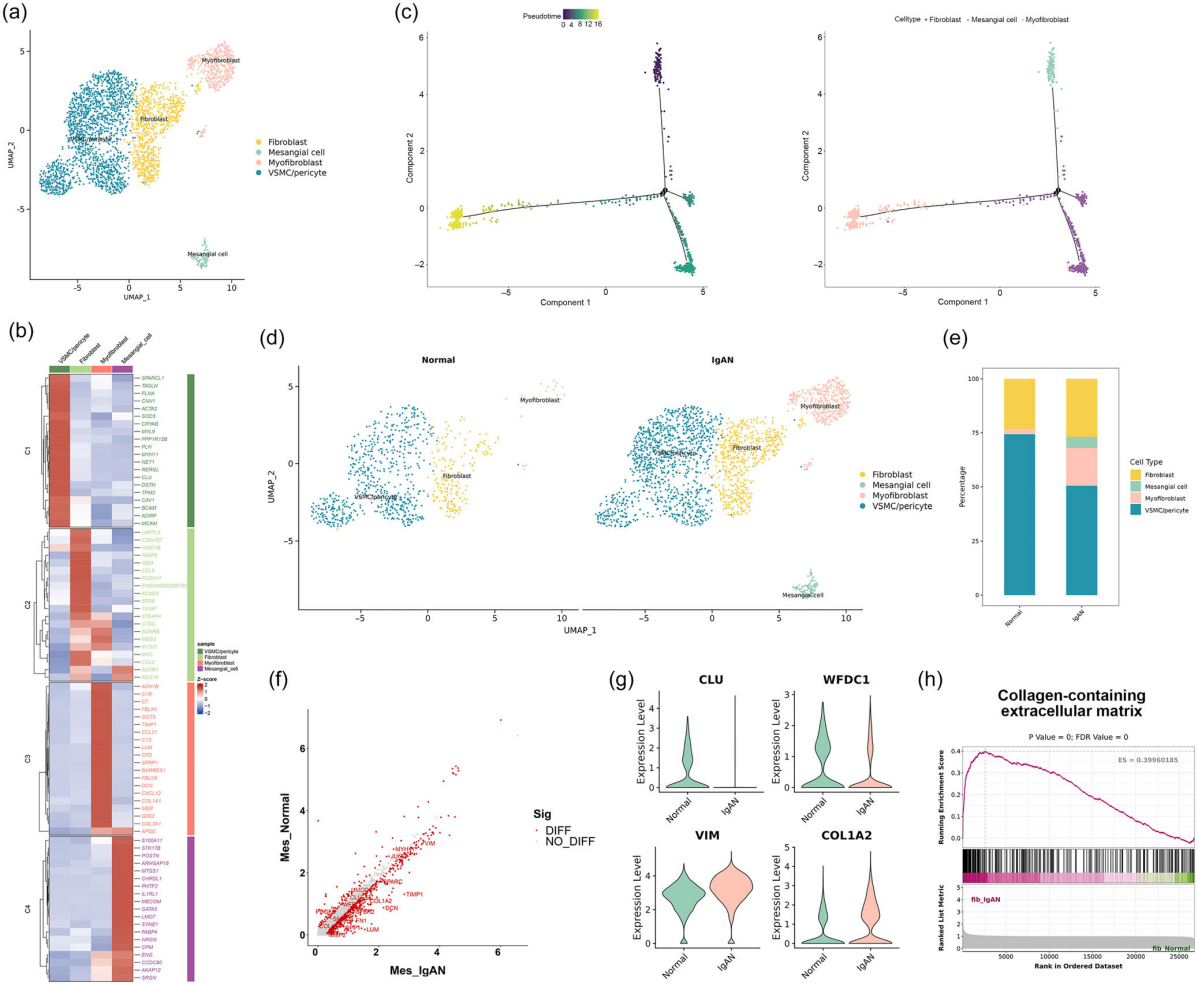
Mesangial and fibroblast characteristics in childhood IgAN. (a) UMAP plot depicted mesangial‐like cell subclusters including mesangial cells, VSMCs/pericytes, fibroblasts and activated myofibroblasts. (b) Heatmap showing the top 20 expressed genes in each subcluster. (c) Pseudotime trajectory analysis for mesangial‐like cell evolution in IgAN. (d,e) Comparison of subcluster percentage in IgAN versus control group. (f,g) Scatter plot (f) and violin plot s(g) howing the DEG between two groups. (h) Enriched KEGG signature of collagen‐containing extracellular matrix for children with IgAN. Abbreviations: DEG, differentially expressed genes; IgAN, IgA nephropathy; KEGG, Kyoto encyclopedia of genes and genomes; UMAP, uniform manifold approximation and projection; VSMC, vascular smooth muscle cell.

Mesangial cells are known to play pivotal role during IgAN pathogenesis; therefore, we initially examined the molecular characterization of this component. According to the gene expression pattern, the mesangial‐like cell cluster was divided into four distinct subgroups, namely true mesangial cells, vascular smooth muscle cells (VSMCs)/pericytes, fibroblasts and activated myofibroblasts (Figure 2a,b). Pseudotime trajectory analysis based on Monocle2 defined distinct states and revealed that differentiation paths of activated myofibroblasts might originate from both fibroblasts and mesangial cells (Figure 2c). Patients with IgAN displayed a remarkably higher proportion of activated myofibroblasts in contrast to non‐IgAN individuals, suggesting active proliferation and phenotype transformation in these subsets of cells (Figure 2d,e). The DEG comparison revealed upregulation of *DCN*, *LUM*, *SPP1*, *VIM* and *COL1A2*, whereas expression of *CLU* and *WFDC1* declined (Figure 2f,g). Gene set enrichment analysis showed an enriched signature of collagen‐containing extracellular matrix in IgAN versus the control group (Figure 2h). These data were in line with the heightened activities of mesangial cells in IgAN pathogenesis.

### 3.2 TREM2^+^ macrophages accumulate in the kidneys of IgAN

**FIGURE 3:**
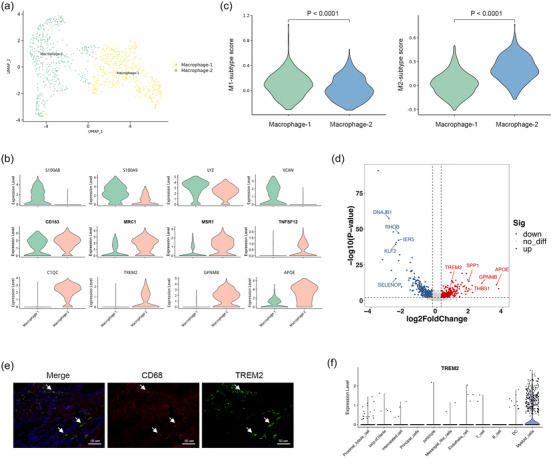
Myeloid cell compositions in childhood IgAN. (a) UMAP plot showing the myeloid cluster of Macrophage‐1 and Macrophage‐2 subgroups. (b) Violin plot depicting expression of the selected marker genes for each subcluster. (c) Comparison of gene score for M1 and M2 macrophage subtypes in Macrophage‐1 versus Macropage‐2. (d) Volcano plot for DEG between IgAN and control groups. (e) Double immunofluorescence staining for TREM2 and CD68. Scale bars: 50 µm. (f) Distribution of TREM2 expression in different cell clusters. Abbreviations: DEG, differentially expressed genes; IgAN, IgA nephropathy; UMAP, uniform manifold approximation and projection.

Macrophages are increasingly recognized as a critical component in regulating the progression of IgAN. To delineate the transformation of macrophages in childhood IgAN, we performed subcluster analysis, and two subpopulations of Macrophage‐1 and Macrophage‐2 were separated (Figure 3a). Macrophage‐1 exhibited a phenotype similar to monocyte‐derived macrophages with high expression of *S100A8*, *S100A9* and *VCAN*, whereas molecular features of the Macrophage‐2 subgroup were consistent with a macrophags phenotype, as shown by expression of *C1QC* and *APOE* (Figure 3b). Macrophages are classically divided into M1 and M2 subtypes, hence we analysed whether the identified subpopulations were correlated with macrophage subtypes based on scoring for the corresponding gene set (Table S1) (Zhang et al., 2020). The results showed that the Macrophage‐2 subset had significantly increased scores for the M2 subtype, such as the high expression of *CD206* (*MRC1*), whereas the score for the M1 subtype was markedly higher in Macrophge‐1 subgroup (Figure 3b,c). Compared with normal control subjects, macrophages of IgAN children showed 645 significantly upregulated genes and 717 significantly downregulated genes (Figure 3d). Importantly, we found that a group of lipid metabolism‐associated genes were enriched for the IgAN group, including *TREM2*, *GPNMB* and *APOE* (Figure 3d). Of note, TREM2 has been found to be crucial in mediating the main functions of macrophages, such as immune modulation, survival and phagocytotic activities. Our immunostaining analysis confirmed heightened infiltration of TREM2^+^ macrophages in kidneys of IgAN (Figure 3e). The antibody used for immunofluorescence was validated, as shown by positive TREM2 expression in THP‐1 cells, a human monocyte line, and negative expression in control non‐immune cell lines (Figure S2a). Additionally, we confirmed that TREM2 was exclusively enriched in cells of the myeloid lineage (Figure 3f). TREM2 expression was also higher in the M2 subtype (Macrophage‐2) cells than in the M1 subtype (Macrophage‐1) (Figure 3b). Therefore, IgAN was characterized by enhanced infiltration of TREM2^+^ macrophages, which implied its potential influence on IgAN pathogenesis.

### 3.3 sTREM2 level is associated with clinical and pathological severity of IgAN

**FIGURE 4:**
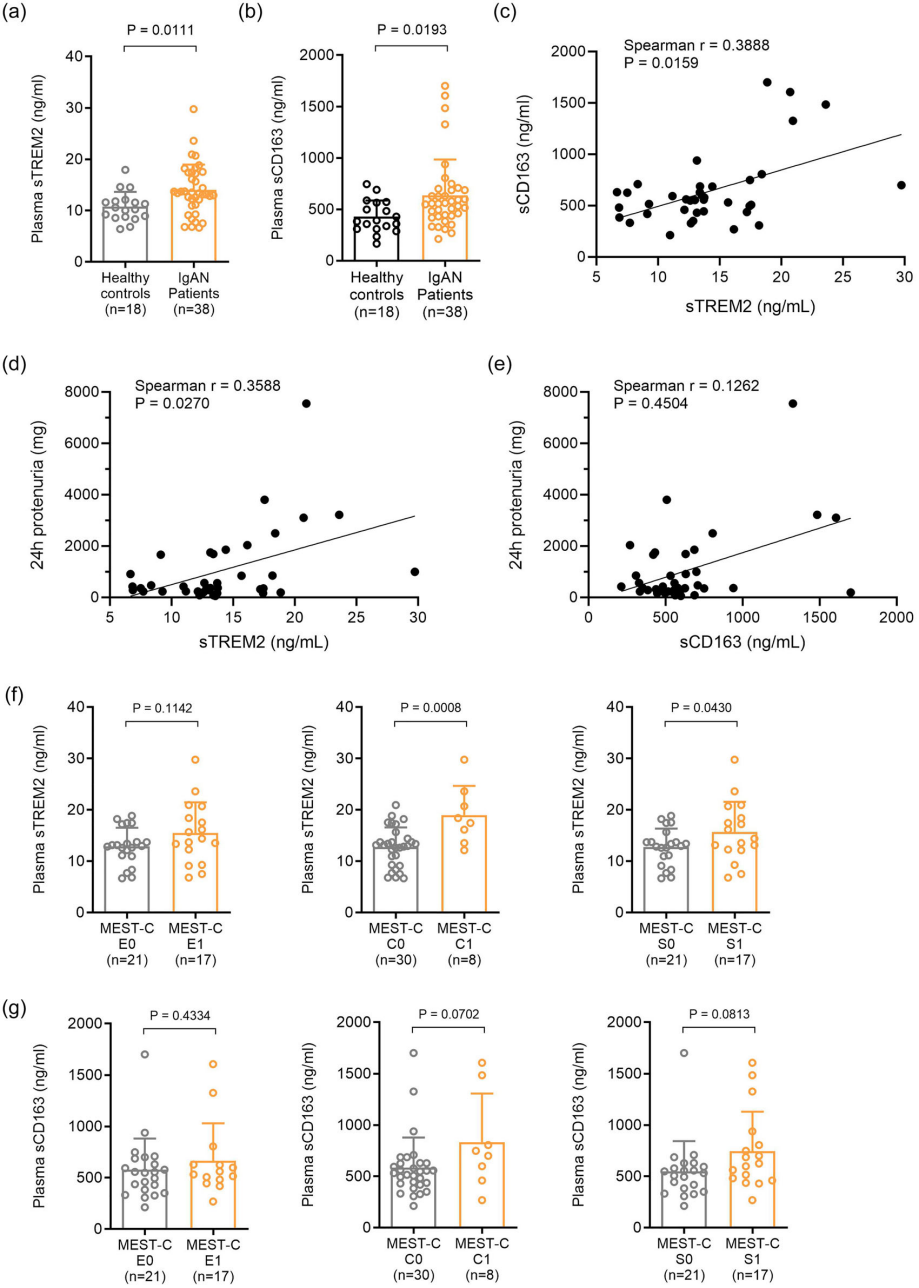
Clinical relevance of sTREM2 in childhood IgAN. (a,b) Comparison of plasma sTREM2 and sCD163 level in IgAN (*n* = 38) versus healthy individuals (*n* = 18). (c) Correlation analysis of plasma sTREM2 and sCD163 (*n* = 38). (d,e) Correlation of sTREM2 and sCD163 with the amount of 24 h proteinuria (*n* = 38). (f,g) Comparision of plasma sTREM2 and sCD163 abundance among IgAN patients with different pathological grading. Data were presented as mean values + SD. Abbreviations: IgAN, IgA nephropathy; sCD163, soluble CD163; sTREM2, soluble TREM2.

Given that TREM2^+^ macrophages accumulated along with IgAN development, we asked whether sTREM2 in body fluids can be adopted in IgAN as a biomarker. To address this possibility, a cohort of 38 treatment‐naive IgAN children were included to measure plasma sTREM2, and its clinical relevance was analysed. The ELISA experiment for sTREM2 was first validated using in vitro cultured cell lines. Although sTREM2 can be detected in conditioned medium from THP‐1 cells, it was barely detected in control cells with negative TREM2 expression (Figure S2b). Compared with healthy individuals (*n* = 18), IgAN patients (*n* = 38) had a significantly higher plasma level of sTREM2 (mean: IgAN vs. control, 14.05 vs. 10.75 ng/mL; Figure 4a). Plasma soluble CD163 (sCD163), another classic marker for macrophages, was also markedly increased in IgAN versus the control group (mean: IgAN vs. control, 636.2 vs. 428.2 ng/mL; Figure 4b). As shown in scRNA‐seq analysis, *CD163* was broadly expressed in different subpopulations of myeloid cells (Figure 3b). There was a significant correlation between plasma sTREM2 and sCD163 abundance (Figure 4c). Importantly, increased sTREM2 levels rather than sCD163 levels were positively associated with the amount of 24 h proteinuria (Figure 4d,e), suggesting that sTREM2 was correlated with disease progression. Children with elevation of sTREM2 also exhibited a trend of worse proteinuria‐to‐creatinine ratio and a decline in estimated glomerular filtration rate (Figure S3). Next, the relationship of sTREM2 to the pathological MEST‐C score was determined. IgAN patients with more advanced lesions of C1 and S1 pathogenic alterations displayed higher sTREM2 levels in peripheral blood than those with C0 and S0 score (Figure 4f). Although sCD163 also tended to be positively associated with pathological severity, the differences were not as significant as for sTREM2 (Figure 4g). Taken together, these findings strongly suggested that sTREM2 constituted a potentially useful biomarker in the evaluation of IgAN progression.

## 4 DISCUSSION

IgAN is a most common type of primary glomerulonephritis among children and adolescents. Childhood IgAN has distinct clinical manifestations, pathological characteristics and prognosis compared with the adult counterpart. Several lines of evidence have elucidated the cellular compositions of IgAN in adults by scRNA‐seq (Chen et al., 2024; Qing et al., 2024; Zheng et al., 2020). However, the pathogenesis of childhood IgAN is less studied and remains unclear. In the present work, we performed scRNA‐seq for kidneys of IgAN children and profiled the cellular landscape. Consistent with the IgAN pathogenesis, single‐cell transcriptomics confirmed mesangial cell and fibroblasts activation, with remarkable accumulation of immune cells. These findings regarding the aberrant microenvironment are in line with the results of scRNA‐seq in adult IgAN, and suggest potential major cell types participating in disease progression.

Stromal remodelling is a universal feature of IgAN. We could discriminate stromal mesangial‐like cells into VSMCs/pericytes, true mesangial cells, fibroblasts and myofibroblasts, as previously indicated (Abedini et al., 2024). Myofibroblasts were almost exclusively found in IgAN kidneys, in contrast to the control group, in which VSMCs and pericytes constituted the majority of stromal cells. It is likely that myofibroblasts could be derived from fibroblasts and mesangial cells based on pseudotime trajectory analysis. DEG analysis revealed that stromal cells in IgAN tended to be enriched with collagen‐containing extracellular matrix, which is in line with development of fibrosis. The direct comparison of mesangial cells between IgAN and healthy individuals was not performed, given the low number of cells available for analysis in the control group.

The presence of macrophages is associated with worse outcomes in IgAN. Many studies have investigated the relationship between macrophage infiltration and the clinical and pathological characteristics of IgAN (Caliskan et al., 2022; Kawasaki, 2022; Wang et al., 2018). It is recognized that the distribution, number and subtypes of macrophages all have a major impact on disease progression (Liu et al., 2023). Increased abundance of macrophages in both the glomerulus and the tubulointerstitial region were correlated with more severe haematuria and MEST‐C score. In contrast, different subclasses of macrophages are believed to be important in IgAN, because heterogenic subpopulations have diverse phenotypes and functions (Hu et al., 2019; Lee et al., 2011). Traditionally, macrophages have been subdivided into M1 and M2 subgroups according to their surface markers and molecular phenotypes. The M1 macrophages contribute to glomerular injury in the early stage of IgAN by enhancing oxidative stress and the pro‐inflammatory response, whereas cells with M2 polarization are believed to facilitate tissue repair and fibrosis in the late stage (Lee et al., 2011). Indeed, various macrophage subpopulations are likely to coexist, and the functional impact depends on the dominating subtype. Here, we found that a group of macrophages displayed a metabolic signature with upregulation of *TREM2*, *GPNMB* and *APOE*, which was also observed in adult IgAN. TREM2 was uniquely expressed on macrophages in IgAN, consistent with results in other organs and disorders. In fact, TREM2^+^ macrophages have been identified in a variety of pathological conditions, with crucial functions, such as mediating efferocytosis and anti‐inflammatory modulation (Wang et al., 2023). The molecular role of TREM2 in IgAN remains unclear and has not yet been examined. In other contexts of experimental chronic or acute kidney injury, TREM2^+^ macrophages conferred protective effects on tissue damage (Cui et al., 2024; Subramanian et al., 2024). Given the potential functions of TREM2^+^ macrophages in secreting anti‐inflammatory cytokines and clearance of apoptotic or necrotic cells, it is presumed that they are able to inhibit IgAN progression. Further clinical and experimental evidence is required to unveil the role of TREM2^+^ macrophages in IgAN.

Predicting IgAN progression is of major clinical importance, because a proportion of patients eventually develop end‐stage renal failure. The commonly adopted strategies include clinical parameters, such as estimated glomerular filtration rate and pathological evaluation. Biomarkers associated with macrophage infiltration and functions are under intense study, given their crucial role in IgAN. One study demonstrated that quantification of macrophages within the kidney was useful in assessing disease severity and predicting treatment response (Xie et al., 2021). CD163 is a classical surface marker of macrophages (Li et al., 2015), and emerging evidence indicated that its soluble form, sCD163, in urine represented a potential non‐invasive biomarker for IgAN activity (Gong et al., 2021; Li et al., 2024). Likewise, TREM2 can also be detected as sTREM2 in body fluids, such as blood plasma. Indeed, sTREM2 has been shown to be a potential biomarker in a variety of disorders, including Alzheimer's disease and non‐alcoholic fatty liver disease (Deczkowska et al., 2020; Filipello et al., 2022). We found that an increased plasma sTREM2 level was associated with severe proteinuria and advanced pathological MEST‐C grade. Besides, the sTREM2 level was also positively associated with the sCD163 level in blood. Mechanistically, sTREM2 is mostly produced by proteolytic cleavage of surface TREM2. As such, increased sTREM2 might be a result of enhanced cleavage and downregulation of TREM2 on macrophages. In turn, upregulation of sTREM2 is supposed to reflect the decline of the protective function for macrophages. In agreement, the downregulation of tissue TREM2 in liver and concomitant elevation of sTREM2 was correlated with the progression of non‐alcoholic steatohepatitis (Wang et al., 2023). Nonetheless, it is also possible that the increased sTREM2 level simply suggests an upregulation of TREM2 expression in IgAN, as for CD163. The underlying mechanisms of sTREM2 generation and its clinical impact in IgAN require further studies and validation in large cohort.

## 5 CONCLUSION

In conclusion, the present study revealed a remarkable remodelling of cellular composition in childhood IgAN, in particular for the stromal compartment. TREM2^+^ macrophages were found to accumulate in the kidney of IgAN patients. We identified that the plasma sTREM2 level was associated with disease severity and therefore constituted a potential non‐invasive biomarker for IgAN.

## AUTHOR CONTRIBUTIONS

Ling Yu and Jianhua Mao conceived the project and designed the experiments. Ling Yu, Xuan Gang and Qiuyu Li collected all the samples. Ling Yu, Jingjing Wang and Guoping Huang analysed all the clinical information. Ling Yu and Xuan Gang performed scRNA‐seq analysis. Weizhong Gu assessed all the histology in this study. Jingjing Wang and Haidong Fu provided useful suggestions for this manuscript. Ling Yu and Jianhua Mao wrote the manuscript. All authors approved the final version of the manuscript and agree to be accountable for all aspects of the work in ensuring that questions related to the accuracy or integrity of any part of the work are appropriately investigated and resolved. All persons designated as authors qualify for authorship, and all those who qualify for authorship are listed.

## CONFLICT OF INTEREST

None declared.

## Supporting information



Supporting Information.

Supporting Information

Supporting Information

Supporting Information

## Data Availability

Requests for scRNA‐seq data and other materials should be addressed to the corresponding author.
