# Supplementary figures and images for: TREM2+ macrophages accumulate in childhood IgA nephropathy and soluble TREM2 represents a reliable non‐invasive biomarker
Source: Exp Physiol. 2025 May 5:10.1113/EP092716. Online ahead of print. doi: 10.1113/EP092716 (PMC13394584; doi:10.1113/EP092716)

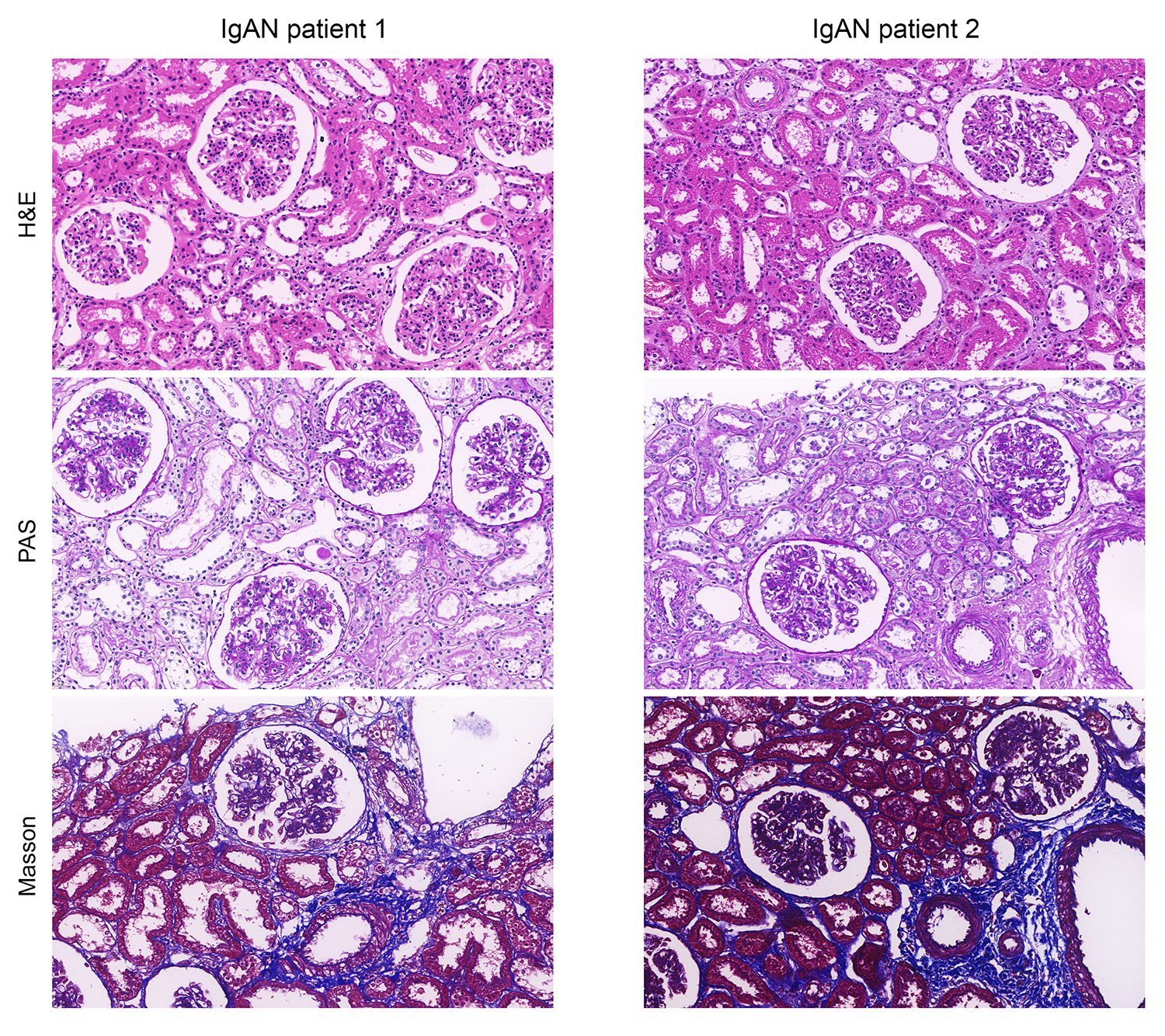

Supplement: Supplementary file 1 — Supporting Information. [file EPH-9999-0-s004.jpg]

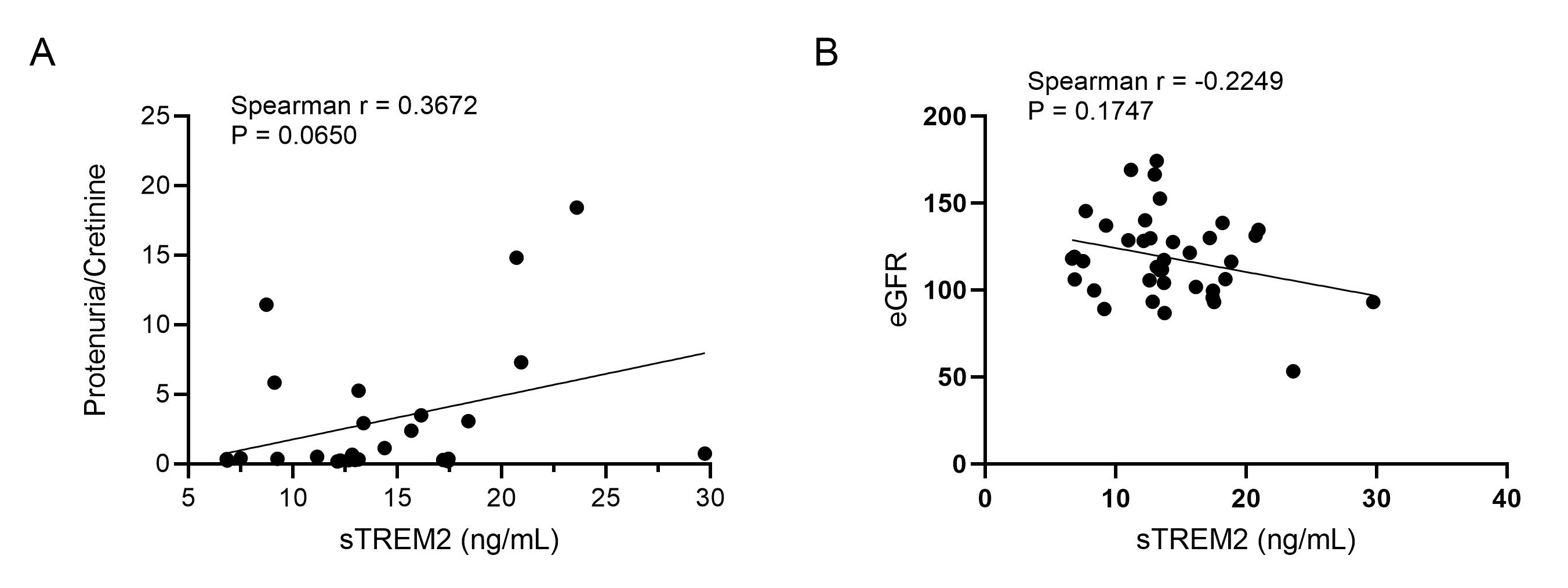

Supplement: Supplementary file 2 — Supporting Information [file EPH-9999-0-s001.jpg]

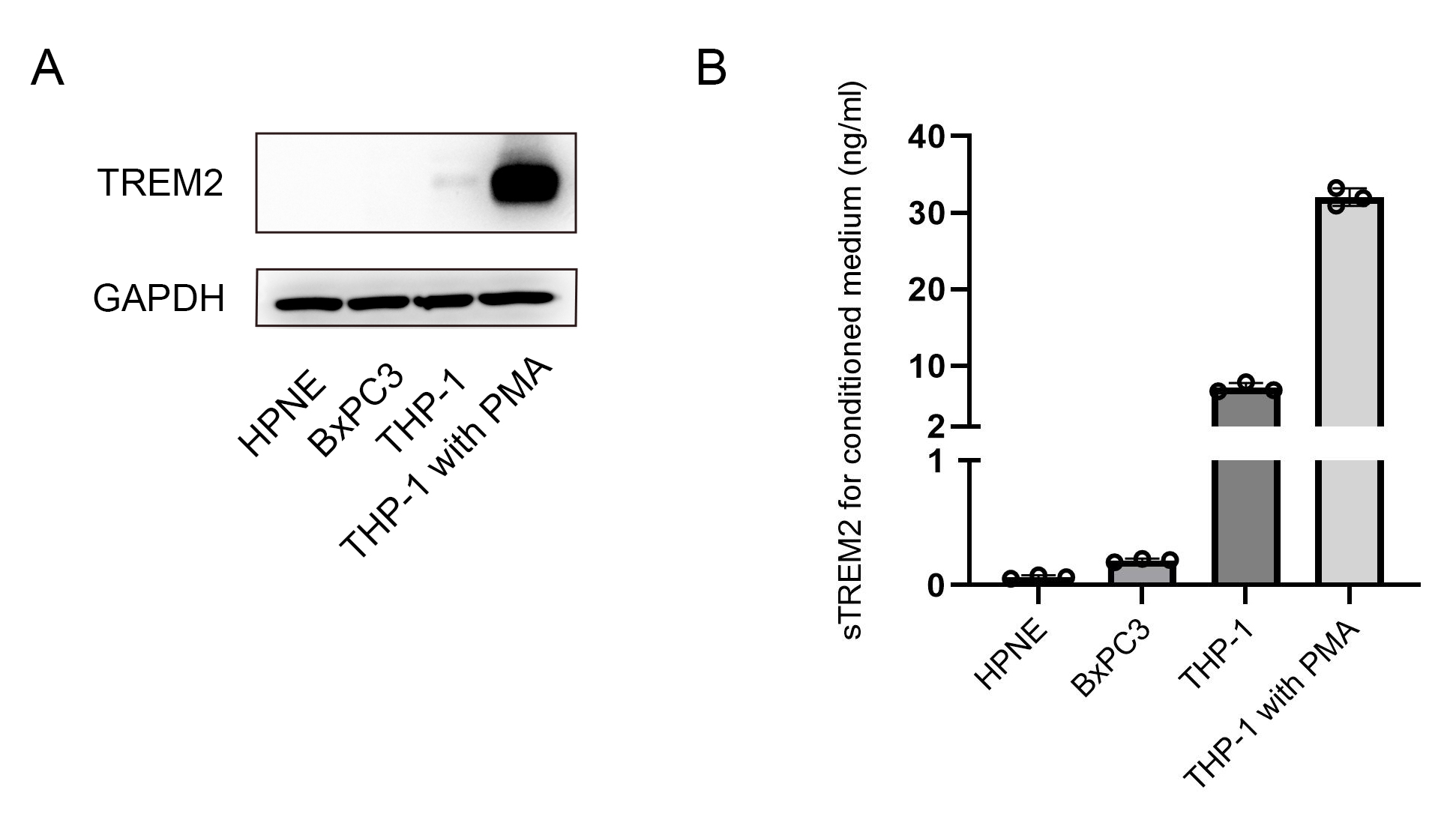

Supplement: Supplementary file 3 — Supporting Information [file EPH-9999-0-s003.jpg]
